# A Forward Genetic Screen and Whole Genome Sequencing Identify Deflagellation Defective Mutants in *Chlamydomonas*, Including Assignment of ADF1 as a TRP Channel

**DOI:** 10.1534/g3.116.034264

**Published:** 2016-08-12

**Authors:** Laura K. Hilton, Fabian Meili, Paul D. Buckoll, Julie C. Rodriguez-Pike, Courtney P. Choutka, Jaime A. Kirschner, Freda Warner, Mette Lethan, Fabian A. Garces, Jingnan Qi, Lynne M. Quarmby

**Affiliations:** Department of Molecular Biology & Biochemistry, Simon Fraser University, Burnaby, British Columbia, V5A 1S6, Canada

**Keywords:** calcium, *Chlamydomonasreinhardtii*, TRP15, FAP16, Mutant Screen Report

## Abstract

With rare exception, ciliated cells entering mitosis lose their cilia, thereby freeing basal bodies to serve as centrosomes in the formation of high-fidelity mitotic spindles. Cilia can be lost by shedding or disassembly, but either way, it appears that the final release may be via a coordinated severing of the nine axonemal outer doublet microtubules linking the basal body to the ciliary transition zone. Little is known about the mechanism or regulation of this important process. The stress-induced deflagellation response of *Chlamydomonas* provides a basis to identifying key players in axonemal severing. In an earlier screen we uncovered multiple alleles for each of three deflagellation genes, *ADF1*, *FA1*, and *FA2*. Products of the two *FA* genes localize to the site of axonemal severing and encode a scaffolding protein and a member of the NIMA-related family of ciliary-cell cycle kinases. The identity of the *ADF1* gene remained elusive. Here, we report a new screen using a mutagenesis that yields point mutations in *Chlamydomonas*, an enhanced screening methodology, and whole genome sequencing. We isolated numerous new alleles of the three known genes, and one or two alleles each of at least four new genes. We identify ADF1 as a TRP ion channel, which we suggest may reside at the flagellar transition zone.

During interphase, basal bodies nucleate the axonemal core of cilia. Although cilia perform a variety of important functions (Quarmby and Leroux 2010; Nachury 2014; Wang and Barr 2016; Morgan and Birman 2016; Hilgendorf *et al.* 2016), they are almost always lost when cells enter mitosis and basal bodies resume their roles as centrosomes (Quarmby and Parker 2005; Kim and Tsiokas 2011; Ke and Yang 2014; Cross and Umen 2015). Cilia are also shed in response to stress, a process known as deflagellation (Craige *et al.* 2013; Marshall 2008; Quarmby 2004). Physiological experiments with the unicellular alga *Chlamydomonas reinhardtii* reveal that axonemal severing is triggered by an influx of Ca^2+^ near the base of the flagella (Quarmby and Hartzell 1994; Quarmby 1996; Wheeler *et al.* 2008). In *Chlamydomonas*, the final event in premitotic ciliary disassembly is axonemal severing at the transition zone (TZ) between the cilium and basal body, similar to the severing that occurs during deflagellation (Parker *et al.* 2010; O’Toole and Dutcher 2014).

In a previous screen for deflagellation-defective mutants, we used insertional mutagenesis to tag affected genes for subsequent cloning and identification (Finst *et al.* 1998). We uncovered 13 mutant strains that resolved into multiple alleles of each of three deflagellation genes: *FA1*, *FA2*, and *ADF1* (Finst *et al.* 1998). *Adf* mutants deflagellate if permeabilized with detergent or dibucaine in the presence of Ca^2+^, indicating that they have functional axonemal severing but are defective in the pathway that activates Ca^2+^ influx (Quarmby and Hartzell 1994; Quarmby 1996). In contrast, *fa* mutants fail to deflagellate even when treated with detergent or dibucaine, indicating defective axonemal severing (Lohret *et al.* 1998). The identity of ADF1 remained elusive, but we identified FA1 as a low complexity scaffolding protein (Finst *et al.* 2000), and FA2 as a NIMA-related kinase (Mahjoub *et al.* 2002). As a Nek kinase, FA2 belongs to an ancient and diverse clade of kinases with dual roles in cilia and cell cycle regulation (Parker *et al.* 2007). FA2 localizes to the TZ both in *Chlamydomonas* cells and when expressed in mammalian cells (Mahjoub *et al.* 2004), providing evidence of a conserved pathway for activating axonemal severing.

We propose that some genes involved in severing the axoneme during deflagellation and preceding mitosis are shared. Thus, although deflagellation itself is not essential to life, genes encoding proteins involved in both processes would be essential. Therefore, the identification of conditional mutants could deepen our understanding of an important premitotic activity.

The advent of whole genome sequencing (WGS) opened the door to identifying conditional mutations in *Chlamydomonas* (Lin and Dutcher 2015). Knowing that we had missed at least two activities (microtubule severing and calcium sensing) in our original screen, we conducted an unbiased forward genetic screen for conditional deflagellation mutants. We report identification of ADF1, discovery of at least four new deflagellation loci, and identification of flagellar protein FAP16 as a player in the signaling pathway.

## Materials and Methods

### Strains and culture conditions

The wild-type 137c mt+ (CC-125) and S1D2 mt− strains (CC-2290) were obtained from the *Chlamydomonas* Resource Center (University of Minnesota). The *Chlamydomonas* bacterial artificial chromosome (BAC) library was from Clemson University Genomics. Cells were maintained on solid tris-acetate-phosphate (TAP; Harris 2009) plates containing 1.5% agar at 21° and under constant illumination. The *adf1*, *fa1*, and *fa2* strains isolated in our previous genetic screen (Finst *et al.* 1998) are also available from the *Chlamydomonas* Resource Center. To induce gametogenesis for mating, cells were maintained on low-N TAP media for 24–48 hr.

### Genetic screen

To mutagenize, 5 × 10^6^ wild-type cells in 20 ml of TAP were irradiated with ultraviolet light for 3 min at 254 nm, then left in the dark for 24 hr to prevent photo-reactivation. They were then plated onto 1.5% agar TAP plates at low density to obtain single colonies. After 4-6 d in continuous light at 21°, individual colonies were picked to agar plates for further growth. To test for deflagellation phenotype, cells were inoculated in 100 µl liquid TAP in 96-well plates and incubated at 21° for 2 d, then transferred to the restrictive temperature of 33° for 6 hr. For manual screening, colonies were screened for acid deflagellation by light microscopy (Finst *et al.* 1998). To make the screening process faster, we introduced a plate reader screen strategy based on Engel *et al.* (2011); absorbance at 510 nm was measured to establish the baseline density of motile cells before treating wells with 1.25 vol of Acid Deflagellation Buffer (ADB; 40 mM sodium acetate, pH 4.5, 1 mM CaCl_2_; Finst *et al.* 1998) for 45 sec, followed by neutralization with 1 vol of 0.1 M NaHCO_3_. Cells were then left to phototax above a light source for 30 min, after which another absorbance reading was taken. The change in absorbance was calculated as follows:

$$\Delta A_{510}=\;\frac{A_{510}{}^{\circ}-A_{510}{}^{\prime }}{A_{510}{}^{\circ}}$$

where $A_{510}{}^{\circ}$ is absorbance before deflagellation and $A_{510}{}^{\prime }$ is absorbance after deflagellation and phototaxis. Each plate had two wells of each of 137c mt+ (fully deflagellated) and an established *adf*-mutant (*adf1-15* E20 G2), and the absorbance readings from these controls were used to establish a cutoff $\Delta A_{510}\;$ for each plate screened to flag colonies for further deflagellation assays. Potential mutants were then rescreened by light microscope manually, as described above. Of the 46,500 total colonies screened for deflagellation, 27,500 were screened using the plate method. The rate of recovery of mutants in the manual screen was about one per 1000 colonies screened, whereas it was only 0.6 per 1000 colonies screened for the plate method. Notably, the plate screening method was sensitive enough to detect a weak conditional mutant, *adf4-1*.

### Mutant classification

In order to classify all mutant isolates as either *adf* or *fa*, cells in liquid culture were treated with 1.25 vol ADB or 1 vol of 150 µM dibucaine on a slide, and fixed with 1 vol of 3% glutaraldehyde after 45 sec. A separate sample of cells was fixed directly in 1 vol of glutaraldehyde to control for the fraction of flagellated cells before deflagellation. To classify mutants as temperature sensitive (ts), cultures of cells were divided and half were incubated at 33° for 6 hr and the other half remained at 21°. Percentage deflagellation was calculated as follows (Finst *et al.* 1998):

$$\%\;\text{deflagellation}=\frac{BFC_{c}-BFC_{e}}{BFC_{c}}\times 100\%$$

where BFC is the number of biflagellate cells per 100 cells, *c* is control, and *e* is experimental.

### PCR-based recombination mapping

To map the causative mutations in each of our new *adf* mutants, we crossed each strain with the polymorphic mapping strain S1D2 (Silflow *et al.* 1995), and used primers from the Kit for Molecular Mapping of *Chlamydomonas* Genes version 1.1 (*Chlamydomonas* Resource Center, University of Minnesota; Rymarquis *et al.* 2005) for PCR-based recombination mapping. Detailed recombination mapping of *adf1* was performed by crossing *adf1;pf16* mutants with S1D2, and identifying recombination events in single mutant progeny using custom primers throughout the *ADF1* region (Kirschner 2009).

### WGS

Genomic DNA was isolated by two cycles of chloroform extraction and ethanol precipitation (Kirschner 2009). For some mutant strains (identified in Supplemental Material, Table S1), at least 5 µg of DNA was submitted to Genewiz (www.genewiz.com) for library preparation and sequencing on a HiSequation 2500 platform in a 2 × 100 bp paired-end (PE) configuration (Table S1). For other strains, genomic DNA libraries were prepared as follows: 5 µg of genomic DNA in 50 µl dH_2_O was fragmented on a Covaris M220 ultrasonicator, using the manufacturer’s 800-bp fragment size protocol. The fragments were dual size selected with a 0.4 vol ratio of beads followed by a 1.0 vol ratio of beads. Fragmented DNA was then ligated to adapters and indexed using the NEBNext Ultra DNA Library Prep Reagent Set for Illumina, as per the manufacturer’s protocol. We monitored DNA fragment size and library concentration with Agilent Bioanalyzer 2100 and Qubit fluorometer (Life Technologies), respectively. For *adf2-1* and *adf5-1*, 0.05 pmol was run on a MiSeq platform using the 300-cycle (150 bp PE) V2 reagent kit. The remaining libraries were sequenced at The Center for Applied Genomics (Hospital for Sick Children, Toronto) on a HiSequation 2500 platform in a 2 × 125 bp PE configuration (Table S1).

The MiSeq and HiSeq outputs were processed as follows: The reads were aligned to the reference genome (*C. reinhardtii* v5.0 assembly; Merchant *et al.* 2007) using Bowtie2 and BWA-MEM, and each analyzed for single nucleotide variants (SNVs) and indels via the Genome Analysis Toolkit (GATK) Haplotypecaller and samtools mpileup (McKenna *et al.* 2010; Li and Durbin 2009; Langmead 2010; Li *et al.* 2009; Li 2011). All four variant (vcf) files were then combined into one, and analyzed for overlapping variant calls. Each vcf file was subtracted against vcf files from other mutant strains to remove parental variants. The BWA-MEM–generated alignments for all *adf* mutants were also analyzed with the variant caller Strelka (Saunders *et al.* 2012), to search for SNVs and small indels, using the sequence from *fa1-6* as the normal sample for subtraction. Variant calls were filtered for homozygous mutations using SnpSift (Cingolani *et al.* 2012a), then annotated with SnpEff (Cingolani *et al.* 2012b) to predict the impact of variants (stop codons, splice site mutations, amino acid substitutions, synonymous mutations, or UTR/intron mutations). The SnpEff reference database was built from the *C. reinhardtii* reference genome and genome annotation v5.5 (Creinhardtii_281_v5.5.gene.gff3.gz; Merchant *et al.* 2007).

To check for large indels and other structural variants, such as duplications, inversions, and translocations, the BWA-MEM alignment for all *adf* mutants were analyzed with the structural variant caller Manta (Chen *et al.* 2016), using the sequence from *fa1-6* as the normal sample for subtraction. Resulting vcf files were input into the Integrated Genomics Viewer (Broad Institute), along with corresponding bam files, to examine the evidence for each predicted structural variant.

### Chlamydomonas transformation

Qiagen Plasmid Midi and Maxi kits were used to prepare plasmid and BAC DNA, respectively. *Chlamydomonas* cells were transformed using the glass bead method (Kindle 1990). For BAC or digested BAC transformations, cells were cotransformed with plasmid pSI103, which confers resistance to paromomycin (Sizova *et al.* 2001). Paromomycin-resistant colonies were screened for rescue of the deflagellation defect, as described in Finst *et al.* (1998).

### PCR confirmation of adf1-3 deletion and rescue

To confirm the putative deletion in *adf1-3* detected by WGS, we designed one primer to anneal within the deletion (GGCCGACATCTACAGGTTGT) and one just downstream of it (GCCTCTCTCACTCCCCCTAC). This primer pair should produce a 2-kb product in strains lacking a deletion. As an internal control, we used primers that amplify IFT20 (forward: TGGACGCGGTAGATAGAGGA, reverse: TCACCTTGATCAGCGACTGC), which produces a 1-kb PCR product.

### Phylogenetic analysis

For the TRP15 phylogenetic analysis, representative transient receptor potential (TRP) channel sequences were obtained for each TRP family and the Shaker K^+^ channel outgroup, according to Peng *et al.* (2015), and included *Chlamydomonas* TRP15, Shaker, and PKD2 sequences. Accession numbers are available in Table S2. Ion transport domains were identified by InterProScan (http://www.ebi.ac.uk/interpro) and aligned with MUSCLE (Edgar 2004). The phylogenetic tree was inferred using maximum likelihood, a WAG+F+G amino acid substitution model, and a bootstrap value of 100, using MEGA7 (Kumar *et al.* 2016).

The FAP16 phylogenetic analysis was completed using phylogeny.fr (Dereeper *et al.* 2008) as follows: Sequences were aligned with MUSCLE (Edgar 2004) and curated to remove gaps using G-blocks (Castresana 2000). The phylogenetic tree was created with PhyML using the WAG amino acid substitution model and an approximate likelihood ratio test (Guindon *et al.* 2010; Anisimova and Gascuel 2006). Trees were rendered using TreeDyn (Chevenet *et al.* 2006). Sequences were obtained by BLAST against human EML1. Accession numbers are available in Table S3.

### Resource and data availability

All new mutant strains have been deposited at the *Chlamydomonas* Resource Center (University of Minnesota). WGS data are available at NCBI BioProject PRJNA338310.

## Results and Discussion

### Forward genetic screen for deflagellation mutants

Following ultraviolet mutagenesis, single colonies were isolated, grown in liquid media, and manually screened for deflagellation defects in response to pH shock, as described in Finst *et al.* (1998). From 46,500 total colonies screened, we uncovered 36 new strains defective in the deflagellation response. The new mutants were classified as *adf* or *fa* on the basis of their deflagellation response to pH shock or dibucaine. Mutants that deflagellate in response to dibucaine but fail to deflagellate in response to pH shock were classified as *adf*, while mutants that fail to deflagellate in response to either treatment were classified as *fa*. Populations of mutants were frequently not uniform in their deflagellation response. Strains that yielded a higher proportion of deflagellating cells at 21° than 33° were categorized as ts. Of the 36 new deflagellation-defective strains, 30 were classified as *adf* and six as *fa*.

### Mapping of mutant strains

**Figure 1:**
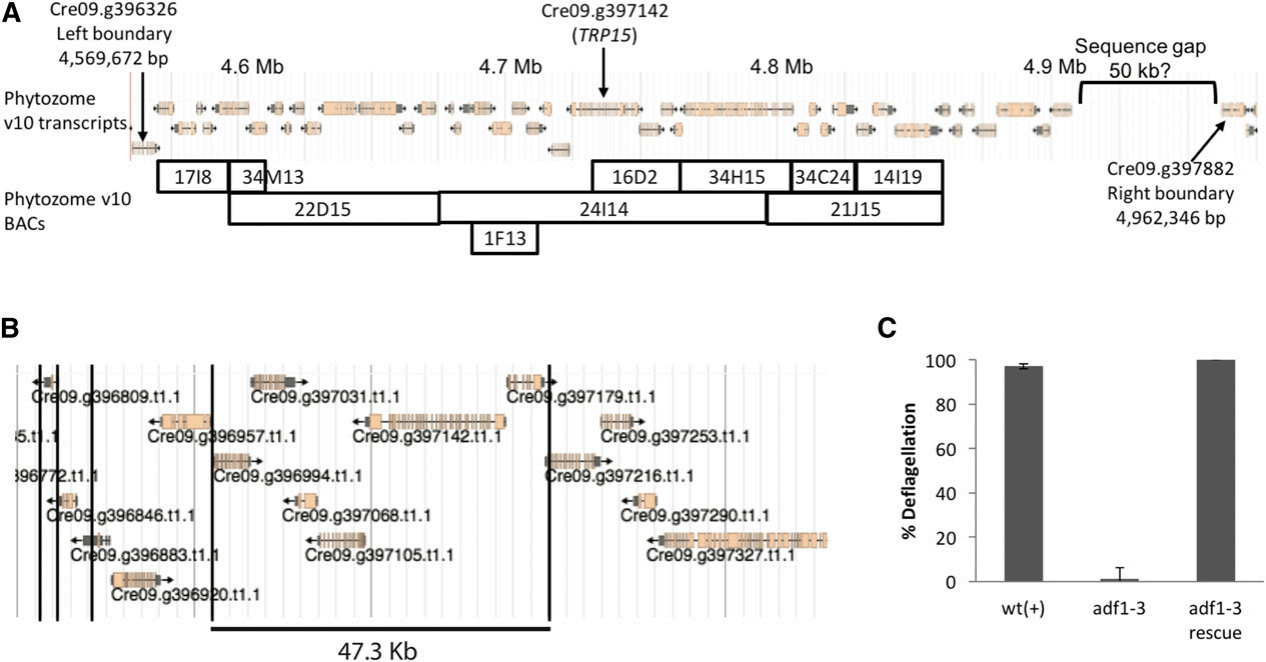
Identification of the *ADF1* gene. (A). A schematic depicting gene predictions within the genomic region known to contain the *ADF1* gene on the basis of recombination mapping. Shown below are the boundaries of BACs spanning this region. (B) A schematic of the genomic region covered by BAC 24I14, which complements the *adf1* mutation, showing gene predictions and *Eco*RI restriction sites (black lines). (C). Deflagellation responses of WT, *adf1-3*, and the strain of *adf1-3* rescued with *Eco*RI-digested BAC 24I14 (*adf1-3R*). Mean of three independent experiments ± SD.

Prior to and in parallel with the screen, we did fine resolution genetic mapping of mutant *adf1* alleles isolated in the screen by Finst *et al.* (1998). Four of the five original *adf1* alleles were generated by insertional mutagenesis, yet none of them retained sequence tags, thereby frustrating identification of the causative mutation. From crosses between *pf16*;*adf1-2* and the mapping strain S1D2, we identified three recombination events between *adf1-2* and *OEE1* out of 920 meioses examined. These data indicate that *ADF1* resides 0.33 map units from *OEE1*. To further define the genomic location of the *ADF1* gene, we performed extensive PCR-based recombination mapping using custom-designed primers (Kirschner 2009). This work located *ADF1* to a 380 kb region containing 42 predicted genes (Figure 1A).

We selected 17 of the 30 new *adf* mutants for PCR-based mapping using markers from the *C. reinhardtii* mapping kit (Table 1; Rymarquis *et al.* 2005). Eleven mutants showed linkage to the marker *PSBO* on chromosome 9 and are hypothesized to be alleles of *adf1*. Other strains were assigned *adf* gene numbers on the basis of linkage to markers on distant regions of the genome. The ts *adf* mutant E4N23 is linked to marker *CNA34* on chromosome 3, and is now known as *adf2-1*; additional mapping with custom primers refined this location (Rodriguez Pike 2013). Two strains, E3.1 P15 B4 (*adf3-1*) and E21 C7 (*adf3-2*), are linked to marker *TUG* on chromosome 6. We hypothesized (and confirm below) that these strains are alleles of the gene we are calling *ADF3*. The mutation in E4.1 P3 H3 (*adf4-1*) is linked to marker *PPX1* on chromosome 9 (see Table 1). We could not establish linkage between the mutant phenotype and any of the mapping kit markers for two of the *adf* strains (E4.1 P1 H3 and E5.1 P8 A12).

**Table 1:** Results of recombination mapping of new *adf* mutant isolates, indicating the recombination frequency between the mutation and the closest mapping kit marker

Isolate ID	Tentative Allele Assignment	Closest Mapping Kit Marker Recombination (Recombinant:Parental)
E1 P19 C5	*adf1-7*	2R:17P marker *PSBO* chromosome 9
E2.2 P2 A12	*adf1-8*	0R:17P marker *PSBO* chromosome 9
E4.2 P4 C12	*adf1-9*	0R:13P marker *PSBO* chromosome 9
E4.2 P5 G9	*adf1-10*	0R:17P marker *PSBO* chromosome 9
E4.2 P10 H9	*adf1-11*	0R:35P marker *PSBO* chromosome 9
E6.1 P3 A4	*adf1-12*	1R:23P marker *PSBO* chromosome 9
E6.1 P3 A7	*adf1-13*	2R:26P marker *PSBO* chromosome 9
E12 M20	*adf1-14*	0R:26P marker *PSBO* chromosome 9
E20 G2	*adf1-15*	2R:28P marker *PSBO* chromosome 9
E21 B5	*adf1-16*	1R:20P marker *PSBO* chromosome 9
E21 C13	*adf1-17*	2R:24P marker *PSBO* chromosome 9
E4 N23	*adf2-1*	45R:106P marker *CNA34* chromosome 3
E3.1 P15 B4	*adf3-1*	10R:26P marker *TUG* chromosome 6
E21 C7	*adf3-2*	19R:69P marker *TUG* chromosome 6
E4.1 P3 H3	*adf4-1*	6R:32P marker *PPX1* chromosome 9
E4.1 P1 H3	Unassigned	Not linked to any markers in mapping kit
E5.1 P8 A12	Unassigned	Not linked to any markers in mapping kit

New *fa* strains were crossed with known *fa* alleles *fa1-4* and *fa2-2*, and the progeny screened for deflagellation. The data in Table 2 suggest that we isolated two new alleles of *fa1* and four new alleles of *fa2*. WGS of several alleles confirmed the presence of mutations in the new alleles of *fa1* and *fa2* (Table 2). Therefore, we did not identify any new *FA* genes through this screen.

**Table 2:** Identification of new *fa* strains as alleles of *fa1* and *fa2*

Strain	x *fa1-4*		x *fa2*-2		Tentative Allele Assignment
	*fa* Progeny	Wild-Type Progeny	*fa* Progeny	Wild-Type Progeny	
E12 AN8	27	0	63	18	*fa1-6*
E15.2 P12 E6	21	0	ND	ND	*fa1-7*
E12 AA8	19	3	76	0	*fa2-5*
E12 AC10	81	29	92	0	*fa2-6*
E12 AE48	11	3	30	0	*fa2-7*
E22 A12	17	9	57	0	*fa2-8*

### Identification of causal mutations by WGS

We performed WGS on 29 strains, including five original *adf1* alleles. We used three different WGS library prep and sequencing strategies: 1) Illumina MiSequation 150 bp PE sequencing using NEBNext Ultra library prep, 2) HiSequation 101 bp PE sequencing prepped in a commercial sequencing lab, or 3) HiSequation 126 bp PE sequencing using NEBNext Ultra library prep.

The MiSeq and HiSeq outputs were processed as follows: Reads were aligned to the reference genome (*C. reinhardtii* v5.0 assembly) and analyzed for SNVs and insertions and deletions (indels) that lay within exons and intron-exon boundaries (*C. reinhardtii* v5.5 annotation). Because the parental strain (CC-125) contained a large number of variants relative to the *Chlamydomonas* reference genome (Lin *et al.* 2013), the list of variants for each mutant strain was compared to two other unlinked mutant strains from our study, and identical variants were removed. In the strains sequenced by HiSeq, ∼20% of the variants are unique to each strain, while MiSeq identified more variants overall, and more unique variants (60–70%), likely due to lower coverage of the genome from MiSeq (Table S1). Lastly, to identify potential causative mutations, we cross-referenced our mapping data with sequencing data (Table 3).

**Table 3:** Proposed causative mutations identified by WGS

Allele Name	Strain ID	Mutation	Gene Description	Gene ID (v5.5)
*adf1-2*	Finst *et al.* 1998	G692fsX714	*TRP15*	Cre09.g396142
*adf1-3*	Finst *et al.* 1998	Δ chromosome 9: 4743360–4745263		
*adf1-4*	Finst *et al.* 1998	Unidentified		
*adf1-5*	Finst *et al.* 1998	Unidentified		
*adf1-6*	Finst *et al.* 1998	A1724D		
*adf1-7*	E1 P19 C5	Unidentified		
*adf1-12*	E6.1 P3 A4	P1133F		
*adf1-13*	E6.1 P3 A7	Unidentified		
*adf1-14*	E12 M20	W560L		
*adf1-15*	E20 G2	L139fsX221		
*adf1-16*	E21B5	Unidentified		
*adf1-17*	E21 C13	P1155L		
*adf3-1*	E3.1 P15 B4	c.2731–1G > A (Splice site acceptor, exon 20)	*FAP16*	Cre06.g303400
*adf3-2*	E21 C7	Y841*		
*fa1-6*	E12 AN8	K1014FS	*FA1*	Cre06.g257600
*fa1-7*	E15.2 P12 E6	c.2610–2A > G (Splice site acceptor, exon 16)		
*fa2-5*	E12 AA8	Q129*	*FA2*	Cre07.g351150
*fa2-6*	E12 AC10	G163R		
*fa2-7*	E12 AE48	G592FS		

The deflagellation defects in E4.1 P1 H3 and E5.1 P8 A12 are not linked to any of the standard mapping kit markers, thereby eliminating potential causative mutations close to any mapping kit markers. This left two candidate mutations for E4.1 P1 H3 and five candidates for E5.1 P8 A12. We designed custom PCR mapping markers for each of the candidate mutations and tested them for linkage to the deflagellation phenotype. None of the candidate mutations were linked to the deflagellation phenotype in either of these strains, thus E4.1 P1 H3 and E5.1 P8 A12 remain unmapped and unidentified.

### ADF1 encodes TRP15, a TRP family Ca^2+^ channel

Of the 42 predicted genes in the known *ADF1* region, 35 are covered by at least one BAC clone (Figure 1A). We transformed *adf1-3* with various BAC clones or single-gene subclones from the known *ADF1* region (Kirschner 2009; Rodriguez Pike 2013). At least 250 transformants of each of the candidate genes were assayed for complementation of the *adf* phenotype. Some of the BACs were digested with restriction enzymes prior to transformation so as to increase the likelihood that certain candidate genes would be incorporated intact (Cerutti *et al.* 1997).

Only one wild-type transformant was obtained, and it was generated by complementation with *Eco*R1-digested BAC 24I14 (PTQ8981), suggesting that *ADF1* might be encoded by one of 11 genes that remained intact on this BAC (Figure 1, B and C). To rule out the possibility that this was a wild-type contaminant or a spontaneous reversion, we back-crossed the rescue strain with a wild-type strain and assayed for the deflagellation phenotype of the progeny. If this were a wild-type contaminant or reversion, then all of the progeny would be wild-type for deflagellation; if this was rescue or suppression, then a quarter of the progeny should exhibit the *adf* phenotype. Consistent with complementation, of the 56 random progeny assayed, 18 (32%) had the *adf* phenotype. Furthermore, crosses between the complemented *adf1-3* strain (*adf1-3R*) and *adf1-1* produced 115 random progeny, of which 64 (56%) were wild-type for deflagellation. Together, these data indicate that the BAC fragment restores wild-type deflagellation to at least two alleles of *adf1*.

**Figure 2:**
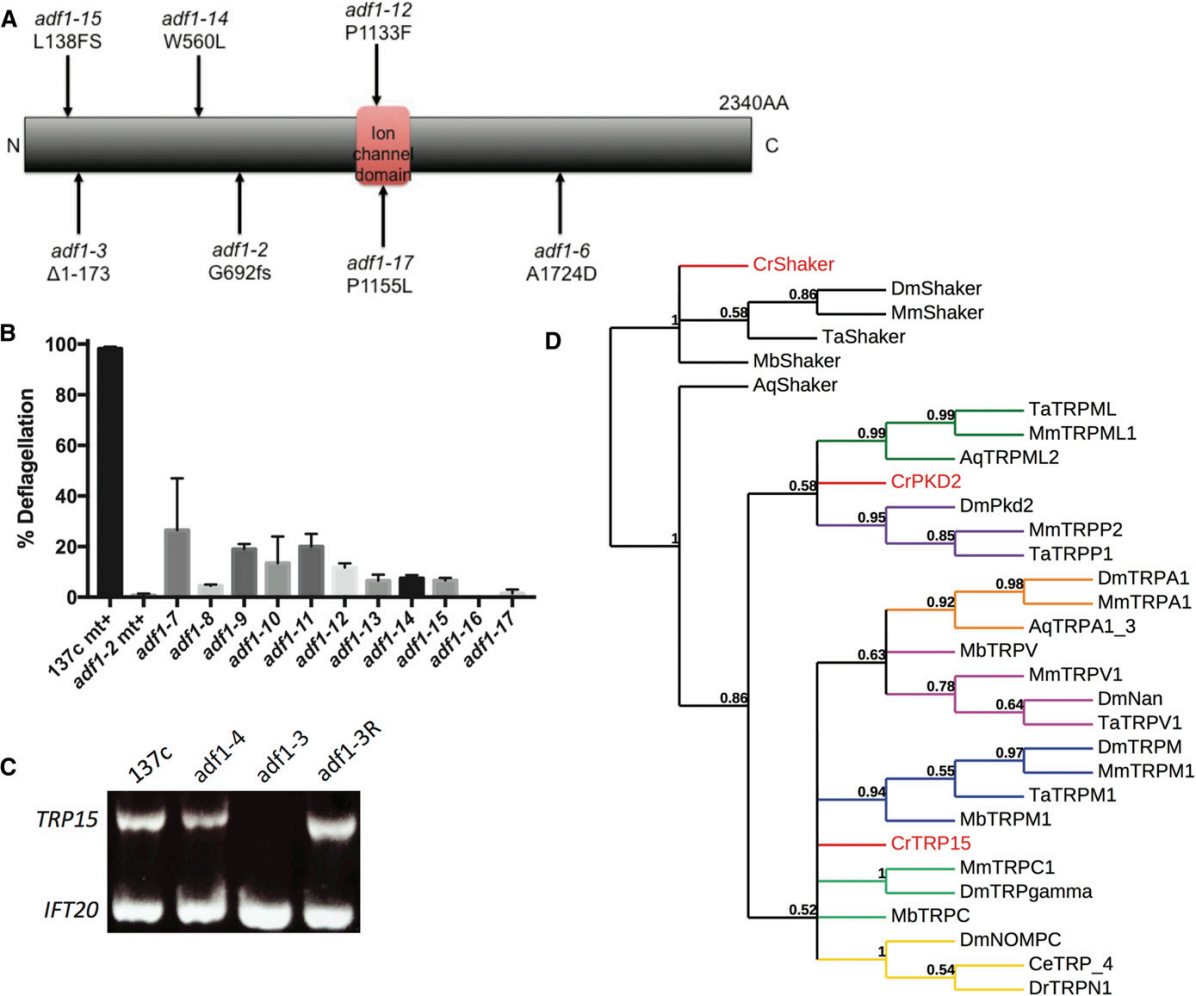
*ADF1* encodes a TRP family ion channel, TRP15. (A) Schematic diagram of TRP15, showing mutation sites in indicated alleles along with the PFAM ion channel prediction. (B) Deflagellation phenotypes of the *adf1* alleles. Each value is the mean of two or more independent experiments ± SEM. (C) PCR confirms the presence of a deletion in *TRP15* in *adf1-3*, which is restored in *adf1-3R*. Of the *TRP15* primers, one anneals within the deleted sequence and the other anneals just upstream of it. *IFT20* was PCR-amplified in the same reaction as a positive control. (D) Phylogenetic analysis placing TRP15 in the context of metazoan TRP clades.

Using WGS, we discovered that seven *adf1* alleles (three from the 1998 collection and four from the new screen) all carry coding mutations in the same gene: *TRP15*, encoding a putative Ca^2+^ channel (Figure 2A and Table 3). All seven of these *adf1* alleles have a deflagellation phenotype similar to that of *adf1-2* (Figure 2B). Using the structural variant caller Strelka, we also identified a 1.9 Kb deletion in *adf1-3*, which deletes all of the 5′ UTR and the first 522 bp of the first exon of TRP15 (Figure 2A and Table 3). We confirmed this deletion by PCR, using a forward primer that anneals inside the putative deletion and one that anneals upstream of it. The deleted DNA was restored when *adf1-3* was rescued by transformation with *Eco*R1-digested BAC 24I14 (Figure 2C). Importantly, the *TRP15* gene is carried intact by an *Eco*R1 fragment of BAC 24I14 (Figure 1B). This supports the idea that restoration of the wild-type deflagellation phenotype of *adf1-3 and adf1-1* was due to rescue and not suppression (Figure 1C).

As described, both WGS and PCR confirm that the missing sequence is restored in the rescue strain, *adf1-3R*. Additionally, WGS coverage for the entire span of the *Eco*RI BAC fragment carrying *TRP15* is 149.2-fold in *adf1-3R* *vs.* 70.0-fold in the flanking sequence, representing a full doubling of sequence coverage for that one region. In *adf1-3*, WGS coverage is equal for the region corresponding to the *Eco*RI fragment relative to the flanking sequence (56.4-fold *vs.* 54.0-fold). We conclude that mutations in *TRP15* cause the deflagellation defect in *adf1* mutants, and that *adf1-3R* is a *bona fide* rescue.

The TRP family is an ancient family of ion channels with diverse functions (Peng *et al.* 2015). We replicated the construction of the phylogenetic tree of TRP channels in Peng *et al.* (2015) and included TRP15 to determine its relationship to other members of the TRP channel family. Based on this analysis, we conclude that TRP15 does not align with any of the metazoan TRP channel clades (Figure 2D). The identification of ADF1 as an ion channel is gratifying, given our earlier data demonstrating that an influx of Ca^2+^ is required to trigger deflagellation (Quarmby and Hartzell 1994; Quarmby 1996).

### ADF3 encodes the flagellar protein FAP16

**Figure 3:**
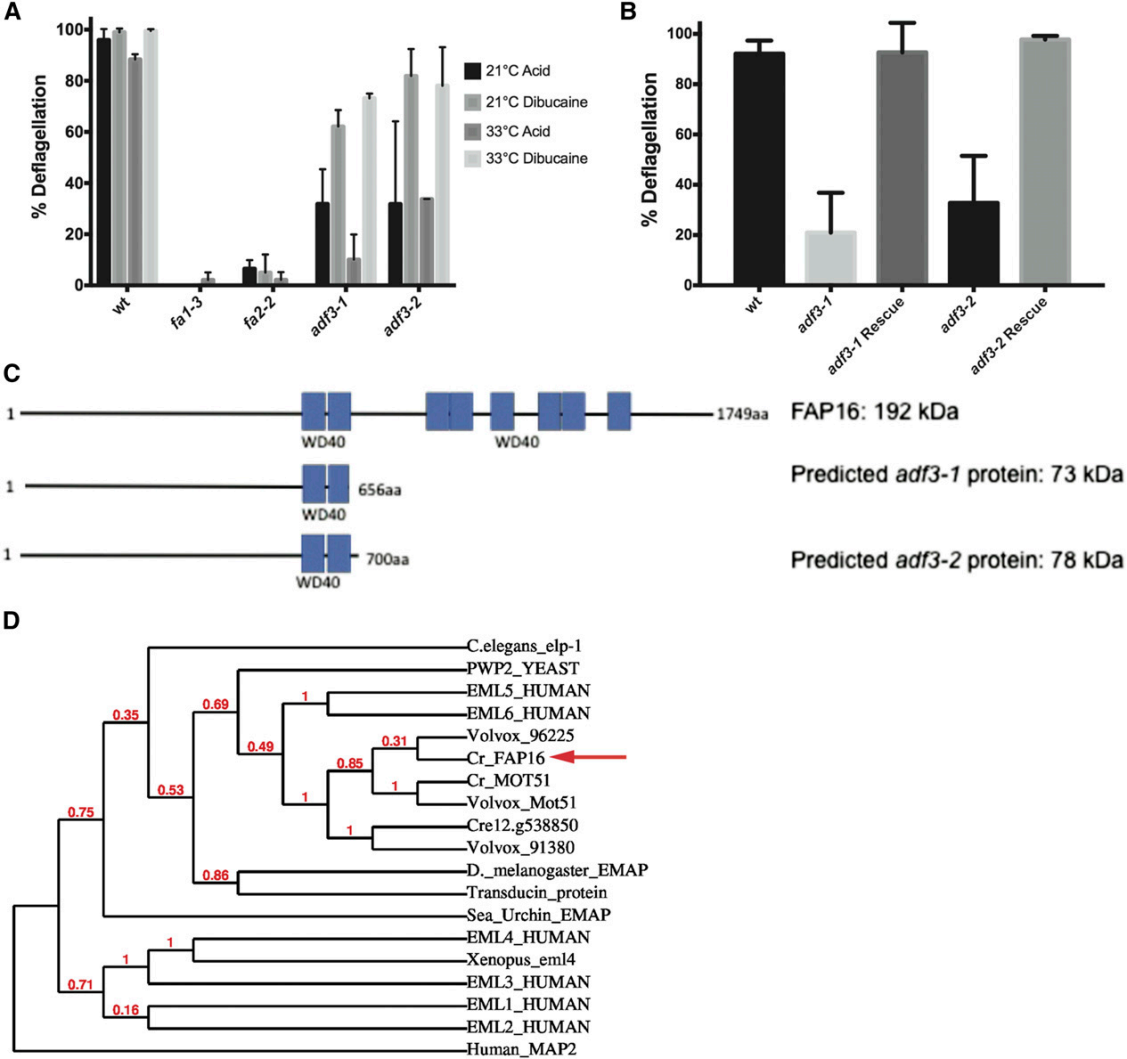
*ADF3* encodes FAP16, a 192 kD flagellar protein. (A) Deflagellation of the newly discovered *adf3-1* and *adf3-2* in response to both dibucaine and acid at 21° and at 33°. Mean of two independent experiments ± SD. (B) Deflagellation response of the two new mutant strains, each transformed with BAC 24K24. Mean of four independent experiments ± SD. (C) Schematic diagram of FAP16 and the predicted truncated protein products of *adf3-1* and *adf3-2*. (D) Phylogenetic tree placing FAP16 in the context of known EML proteins.

Strains *adf3-1* and *adf3-2* have strong defects in acid-induced deflagellation, but are almost wild type in their response to dibucaine (Figure 3A). WGS identified mutations in *FAP16* in both alleles (Table 3), 535 kb from the mapping kit marker *TUG*, which had 22% recombination with the deflagellation phenotype in *adf3-2*. To confirm this gene assignment, *adf3-1* and *adf3-2* cells were transformed with BAC 24K24 (PTQ9022), and we recovered one complemented strain with wild-type deflagellation for each of the two alleles (200 transformants screened for each strain; Figure 3B). In the region covered by BAC 24K24 (73 kb containing 10 predicted genes), WGS coverage for *adf3-1* is 14.5-fold (obtained by MiSeq, Q-score ≥ 10), while coverage for *adf3-2* is 35.1-fold (obtained by HiSeq Q-score ≥ 10). We manually examined the region covered by this BAC for insertions or deletions. In addition to the original analysis with samtools mpileup and GATK HaplotypeCaller, we looked for variants using Strelka and the structural variant caller Manta. We found no other variants of any kind in either of the alleles in the region covered by BAC 24K24, and *FAP16* is the only gene in the region with variants in both *adf3-1* and *adf3-2*. We conclude that *ADF3* is *FAP16*.

FAP16 is a protein of unknown function that was identified in the flagellar proteome of *Chlamydomonas* (Pazour *et al.* 2005). It encodes a 192 kD protein annotated with eight WD40 domains. In *adf3-1*, the causative mutation is a C > G transversion resulting in the loss of a splice site before exon 19; in *adf3-2*, the causative mutation is Y841*. These mutations are predicted to truncate the protein to 73 and 78 kD, respectively (Figure 3C).

WD40 domains are common among diverse proteins, serving generally as sites of protein–protein interactions (Stirnimann *et al.* 2010). BLAST analysis indicates that FAP16 is most similar to a family of microtubule-binding proteins, known as EMLs. Members of the EML family have been shown to be required for microtubule formation and have been implicated in ciliopathies (Lyman and Chetkovich 2015; Pollmann *et al.* 2006). Our phylogenetic analysis suggests that of the human EMLS, FAP16 is most closely related to EML5 and EML6 (Figure 3D). As a flagellar- and possibly microtubule-associated protein with a signaling (*adf*) rather than severing (*fa*) phenotype, FAP16 may serve to localize signaling proteins to the site of severing. The site of axonemal severing defines the juncture between the flagellum and the TZ. The deflagellation protein FA2 localizes to this site and a fraction of the FA2 leaves with the flagella when they are shed (Mahjoub *et al.* 2004). We speculate that FAP16 might similarly localize.

### ADF2 and ADF4 remain unassigned

**Figure 4:**
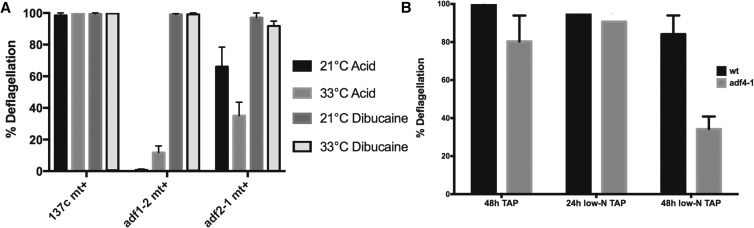
The deflagellation phenotypes of *adf2-1* and *adf4-1* are conditional on temperature and nutrient status, respectively. (A) Deflagellation responses of the newly discovered *adf2-1* strain, at both 21° and 33°. Mean of three independent experiments ± SD. (B) Deflagellation response of *adf4-1* after 24 and 48 hr in low-N TAP media. Mean of four independent experiments ± SD.

The *adf2-1* strain is the only mutant we recovered with a ts deflagellation phenotype (Figure 4A). Wild-type cells deflagellate completely in response to pH shock or dibucaine, regardless of the temperature, and *adf1-2* cells do not deflagellate in response to pH shock at either temperature. In contrast, *adf2-1* mutant cells deflagellate much more in response to pH shock at the permissive temperature than at the restrictive temperature.

We mapped the *adf2-1* mutation to chromosome 3, but the combination of mapping and WGS (by MiSeq) did not provide sufficient resolution for us to assign the gene. One attractive candidate for the causal mutation is W71* in an inositol 1,3,4-trisphosphate 5/6 kinase (*ITPK1*; Cre03.g182100). In *Arabidopsis*, the related enzyme ITPK4 acts in the main pathway to IP6 (phytic acid) synthesis, impacting both signaling pathways and calcium storage (Kim and Tai 2011). Intriguingly, the knockdown of *IPK1*, the IP5-phosphorylating enzyme in zebrafish, reduces ciliary length and motility, linking a ciliary phenotype to a reduction in this inositol phospholipid kinase (Sarmah *et al.* 2007). Nevertheless, we cannot yet assign this gene as *ADF2* because we have only isolated a single allele of *adf2*, and we have not shown tight linkage between the phenotype and this mutation.

In working with *adf4-1* cultures, we observed that the deflagellation phenotype was often weak. Serendipitously, we discovered that cultures maintained in liquid TAP for long periods (≥5 d) had a stronger deflagellation-defective phenotype than freshly inoculated cultures. We hypothesized that nutrient starvation, possibly nitrogen starvation, was responsible for this effect. To test this, we inoculated *adf4-1* and wild-type strains in liquid TAP medium for 48 hr, then treated them in one of three ways: fresh liquid TAP for 48 hr; fresh TAP for 24 hr followed by 24 hr in low-N tap; or low-N liquid TAP for 48 hr. The acid-deflagellation defect was strongest after 48 hr low-N liquid TAP (Figure 4B). Discovery of a nutrient-based conditionality is novel in the deflagellation pathway and it is yet to be determined whether this phenotype is related to gametogenesis.

We mapped *adf4-1* to a region of chromosome 9 distinct from the *ADF1* region (see Table 1). Examination of our WGS data in this region led us to identify a candidate for causal mutation: D780N missense mutation in Cre09.g386732, annotated as a glycosyltransferase. We refrain from assigning this as *ADF4* for the following reasons: we have identified only the one allele, we have not established tight linkage between the mutation and the phenotype, and the D780 is not a conserved amino acid. Therefore, *ADF4* remains unidentified.

### Conclusions

We report at least five new deflagellation genes, map four of them, and identify two. *ADF1* is revealed as a TRP channel and *ADF3* as flagellar protein FAP16. We used genetic mapping and WGS to identify candidate mutations. Gene assignments were confirmed by complementation of the deflagellation phenotype with the wild-type gene.

Acid-induced deflagellation is triggered when weak organic acids freely permeate the cell membrane (Hartzell *et al.* 1993). Inside the cell, dissociation leads to acidification of the cytoplasm and localized Ca^2+^ influx near the base of the flagella (Quarmby 1996; Wheeler *et al.* 2008). We propose that TRP15 is the acid-activated entry route for calcium. In response to the Ca^2+^ signal, microtubules are severed at a precise site at the base of the flagella, distal to the flagellar TZ. We propose that FAP16 connects the signaling components to the microtubule-severing machinery. We have previously localized FA2 to this highly specific location, and predict that FAP16 will similarly localize (both FAP16 and FA2 are found in the flagellar proteome), possibly serving to dock the still-elusive calcium-sensing protein and/or the TRP15 channel. Indirect evidence suggests that the acid-stimulated influx of Ca^2+^ might not directly activate axonemal severing. Instead, the calcium influx might stimulate release of an internal store of Ca^2+^, which in turn triggers severing (reviewed in Quarmby 2009).

The current screen was not saturated: we isolated multiple new alleles of each of the three known deflagellation genes, but only two alleles of *ADF3*, single alleles for each of *ADF2* and *ADF4*, and no new *FA* genes. This latter observation indicates that we still have not uncovered the microtubule-severing protein responsible for axonemal severing. It is also notable that we have yet to identify a calcium-binding protein to activate axonemal severing in response to the calcium signal. Intriguingly, only one of the new strains has a ts deflagellation defect (*adf2-1*), while another has a deflagellation defect that is conditional on nutrient status (*adf4-1*).

More genes remain to be discovered in the deflagellation pathway of *Chlamydomonas*, genes potentially also involved in the premitotic release of centrosomes. While our screen and subsequent mapping and complementation identified several interesting new genes to investigate, a future screen of ts lethal mutant strains could uncover important genes involved in both deflagellation and mitotic progression.

## 

## Supplementary Material

Supplemental Material
