# Supplementary material for: A Forward Genetic Screen and Whole Genome Sequencing Identify Deflagellation Defective Mutants in Chlamydomonas, Including Assignment of ADF1 as a TRP Channel
Source: G3 (Bethesda). 2016 Aug 12;6(10):3409–18. doi: 10.1534/g3.116.034264 (PMC5068960; doi:10.1534/g3.116.034264)
Supplement: Supplemental Material [file supp_6_10_3409__index.html]

A Forward Genetic Screen and Whole Genome Sequencing Identify Deflagellation Defective Mutants in Chlamydomonas, Including Assignment of ADF1 as a TRP Channel — Supplemental Material 

# A Forward Genetic Screen and Whole Genome Sequencing Identify Deflagellation Defective Mutants in *Chlamydomonas*, Including Assignment of ADF1 as a TRP Channel

## Supplemental Material for Hilton, *et al*, 2016

**Files in this Data Supplement:**

- Table S1 - Summary of sequencing results from WGS. (.pdf, 67 KB)
- Table S2 - Accession numbers for genes used in phylogenetic analysis of TRP15. (.pdf, 97 KB)
- Table S3 - Accession numbers for proteins used in phylogenetic analysis of FAP16. (.pdf, 64 KB)
