# Supplementary material for: A Forward Genetic Screen and Whole Genome Sequencing Identify Deflagellation Defective Mutants in Chlamydomonas, Including Assignment of ADF1 as a TRP Channel
Source: G3 (Bethesda). 2016 Aug 12;6(10):3409–18. doi: 10.1534/g3.116.034264 (PMC5068960; doi:10.1534/g3.116.034264)
Supplement: Supplemental Material [file supp_g3.116.034264_TableS1.pdf]

SUPPLEMENTAL MATERIAL

Table S1: Summary of sequencing results from WGS.

Sample	Sequencing Type	Read Length (bp)	Fold Coverage	Mean Q-score	Total Variants	Unique Variants	%Unique Variants
adf1-2	HiSeq	126	27.9	35.84	232411	5952	2.6
adf1-3	HiSeq	126	73.9	35.82	255376	21892	8.6
adf1-3R	HiSeq	101	39.1	34.89	264015	49431	18.7
adf1-3R	HiSeq	126	56.1	35.20	314922	85617	27.2
adf1-4	HiSeq	126	89.7	34.72	237680	14070	5.9
adf1-5	HiSeq	126	71.2	34.97	234785	11447	4.9
adf1-6	HiSeq	126	51.8	35.60	257873	32072	12.4
adf1-7	HiSeq	126	57.0	34.94	10562	2382	22.6
adf1-12	HiSeq	126	76.4	35.00	4541	965	21.3
adf1-13	HiSeq	126	48.8	35.55	9726	2221	22.8
adf1-14	HiSeq	101	45.9	33.23	8072	1287	15.9
adf1-15	HiSeq	126	70.2	35.60	10762	1836	17.1
adf1-16	HiSeq	101	34.4	34.01	8537	1887	22.1
adf1-17	HiSeq	126	76.2	34.00	4703	876	18.6
adf2-1	MiSeq	151	21.7	33.99	7190	1891	26.3
adf3-1	MiSeq	151	15.9	34.15	7340	2758	37.6
adf3-2	HiSeq	101	33.3	33.89	8554	1577	18.4
adf4-1	HiSeq	101	31.0	34.15	8449	2035	24.1
E4.1 P1 H3	HiSeq	101	32.5	34.23	8414	1799	21.4
E5.1 P8 A12	HiSeq	101	35.0	34.08	8725	1833	21.0
fa1-6	HiSeq	126	73.3	34.76	4366	840	19.2
fa1-7	HiSeq	101	34.9	34.07	12607	2460	19.5
fa2-5	HiSeq	126	67.6	34.76	4730	938	19.8
fa2-6	HiSeq	126	69.7	34.58	4586	831	18.1
fa2-7	HiSeq	126	72.5	34.66	4406	764	17.3
