# Supplementary material for: A Forward Genetic Screen and Whole Genome Sequencing Identify Deflagellation Defective Mutants in Chlamydomonas, Including Assignment of ADF1 as a TRP Channel
Source: G3 (Bethesda). 2016 Aug 12;6(10):3409–18. doi: 10.1534/g3.116.034264 (PMC5068960; doi:10.1534/g3.116.034264)
Supplement: Supplemental Material [file supp_g3.116.034264_TableS2.pdf]

**Table S2.** Accession numbers for genes used in phylogenetic analysis of TRP15.

<table>
<tr><th></th><th>M. musculus</th><th>D. melanogaster</th><th>A. queenslandica</th><th>T. adhaerens</th><th>M. brevicollis</th><th>C. elegans</th><th>D. rerio</th><th>C. reinhardtii</th></tr>
<tr><td rowspan="2">TRPN</td><td></td><td>DmNOMPC</td><td></td><td></td><td></td><td>CeTRP-4</td><td>DrTRPN1</td><td></td></tr>
<tr><td></td><td>NP_523483.2</td><td></td><td></td><td></td><td>CAC14420.3</td><td>NP_899192.1</td><td></td></tr>
<tr><td rowspan="2">TRPA</td><td>MmTRPA1</td><td>DmTRPA1</td><td>AqTRPA1-3</td><td></td><td>MbTRPA1-1</td><td></td><td></td><td></td></tr>
<tr><td>NP_808449.1</td><td>NP_648263.5</td><td>XP_003388066</td><td></td><td>jgi|Monbr1|22693</td><td></td><td></td><td></td></tr>
<tr><td rowspan="2">TRPM</td><td>MmTRPM1</td><td>DmTRPM</td><td></td><td>TaTRPM1</td><td>MbTRPM1</td><td></td><td></td><td></td></tr>
<tr><td>NP_001034193.2</td><td>NP_001036548.1</td><td></td><td>jgi|Triad1|58797</td><td>jgi|Monbr1|27446</td><td></td><td></td><td></td></tr>
<tr><td rowspan="2">TRPML</td><td>MmTRPML1</td><td>DmTRPML</td><td>AqTRPML2</td><td>TaTRPML</td><td>MbTRPML</td><td></td><td></td><td></td></tr>
<tr><td>NP_444407.1</td><td>NP_649145.1</td><td>XP_0011402441.1</td><td>jgi|Triad1|27592</td><td>jgi|Monbr1|5128</td><td></td><td></td><td></td></tr>
<tr><td rowspan="2">TRPC</td><td>MmTRPC1</td><td>DmTRPgamma</td><td></td><td></td><td>MbTRPC</td><td></td><td></td><td></td></tr>
<tr><td>NP_035773.1</td><td>NP_609802.1</td><td></td><td></td><td>jgi|Monbr1|27307</td><td></td><td></td><td></td></tr>
<tr><td rowspan="2">TRPV</td><td>MmTRPV1</td><td>DmNan</td><td></td><td>TaTRPV1</td><td>MbTRPV</td><td></td><td></td><td></td></tr>
<tr><td>NP_001001445.1</td><td>NP_001261833.1</td><td></td><td>jgi|Triad1|58478</td><td>jgi|Monbr1|22556</td><td></td><td></td><td></td></tr>
<tr><td rowspan="2">TRPP</td><td>MmTRPP2</td><td>DmPkd2</td><td></td><td>TaTRPP1</td><td></td><td></td><td></td><td>CrPKD2</td></tr>
<tr><td>NP_032887.3</td><td>NP_609561.2</td><td></td><td>jgi|Triad1|28998</td><td></td><td></td><td></td><td>Cre17.g715300</td></tr>
<tr><td rowspan="2">Shaker</td><td>MmShaker</td><td>DmShaker</td><td>AqShaker</td><td>TaShaker</td><td>MbShaker</td><td></td><td></td><td>CrShaker</td></tr>
<tr><td>|NP_067250.2</td><td>CAA29917.1</td><td>XP_011404770.1</td><td>jgi|Triad1|2482</td><td>jgi|Monbr1|8918</td><td></td><td></td><td>Cre10.g432550</td></tr>
<tr><td rowspan="2"></td><td></td><td></td><td></td><td></td><td></td><td></td><td></td><td>CrTRP15</td></tr>
<tr><td></td><td></td><td></td><td></td><td></td><td></td><td></td><td>Cre09.g397142</td></tr>
</table>
