# Supplementary material for: A Forward Genetic Screen and Whole Genome Sequencing Identify Deflagellation Defective Mutants in Chlamydomonas, Including Assignment of ADF1 as a TRP Channel
Source: G3 (Bethesda). 2016 Aug 12;6(10):3409–18. doi: 10.1534/g3.116.034264 (PMC5068960; doi:10.1534/g3.116.034264)
Supplement: Supplemental Material [file supp_g3.116.034264_TableS3.pdf]

Table S3. Accession numbers for proteins used in phylogenetic analysis of FAP16.

Gene Name	Accession Number
EML1_Human	NP_004425
EML2_Human	NP_036287
EML3_Human	NP_694997
EML4_Human	EAX00324
EML5_Human	NP_899243
EML6_Human	NP_001034842
Cr_MOT51	Cre03.g178050
Cr_FAP16	Cre06.g303400
Cre12.g538850	Cre12.g538850
Xenopus eml4	F6SW47
Sea Urchin EMAP	NP_999633
Drosophila EMAP	NP_001261850
VOLCADRAFT_96225	D8U9J7
Volvox MOT51	XP_002946691
VOLCADRAFT_91380	D8TWX1
C. elegans elp-1	NP_001256950
Human MAP2	NP_002365
Transducin	XP_002885447
PWP2_Yeast	NP_009984
